# “I feel drug resistance testing allowed us to make an informed decision”: qualitative insights on the role of HIV drug resistance mutation testing among children and pregnant women living with HIV in western Kenya

**DOI:** 10.1186/s12913-023-09804-x

**Published:** 2023-08-24

**Authors:** Andrea J. Scallon, Shukri A. Hassan, Shirley Rui Qian, Yuandi Gao, Patrick Oyaro, Evelyn Brown, James Wagude, Irene Mukui, Eunice Kinywa, Frederick Oluoch, Francesca Odhiambo, Boaz Oyaro, Leonard Kingwara, Nashon Yongo, Enericah Karauki, Lindah Otieno, Grace C. John-Stewart, Lisa L. Abuogi, Rena C. Patel

**Affiliations:** 1 Jackson School of International Studies, University of Washington, Seattle, USA; 2 Department of Medicine, University of Washington, 325 9th Ave, Seattle, WA 98105 USA; 3 School of Public Health, University of Washington, Seattle, USA; 4 Health Innovations Kenya (HIK), Kisumu, Kenya; 5 UW Kenya, Nairobi, Kenya; 6 Department of Health, Siaya County, Kenya; 7 Drugs for Neglected Diseases Initiative, Nairobi, Kenya; 8 Department of Health, Kisumu County, Kenya; 9 Family AIDS Care and Education Services, Kenya Medical Research Institute, Kisumu, Kenya; 10 Kenya Medical Research Institute-CDC, Kisian, Kenya; 11 National HIV Reference Laboratory, Ministry of Health, Nairobi, Kenya; 12 Departments of Global Health, University of Washington, 325 9th Ave, WA 98105 Seattle, USA; 13 Department of Pediatrics, University of Washington, Seattle, USA; 14 Department of Epidemiology, University of Washington, Seattle, USA; 15 Department of Pediatrics, University of Colorado, Denver, USA

**Keywords:** Viral suppression, Viral load testing, Drug resistance testing, Drug resistance mutation

## Abstract

**Background:**

Pregnant women and children living with HIV in Kenya achieve viral suppression (VS) at lower rates than other adults. While many factors contribute to these low rates, the acquisition and development of HIV drug resistance mutations (DRMs) are a contributing factor. Recognizing the significance of DRMs in treatment decisions, resource-limited settings are scaling up national DRM testing programs. From provider and patient perspectives, however, optimal ways to operationalize and scale-up DRM testing in such settings remain unclear.

**Methods:**

Our mixed methods study evaluates the attitudes towards, facilitators to, and barriers to DRM testing approaches among children and pregnant women on antiretroviral therapy (ART) in five HIV treatment facilities in Kenya. We conducted 68 key informant interviews (KIIs) from December 2019 to December 2020 with adolescents, caregivers, pregnant women newly initiating ART or with a high viral load, and providers, laboratory/facility leadership, and policy makers. Our KII guides covered the following domains: (1) DRM testing experiences in routine care and through our intervention and (2) barriers and facilitators to routine and point-of-care DRM testing scale-up. We used inductive coding and thematic analysis to identify dominant themes with convergent and divergent subthemes.

**Results:**

The following themes emerged from our analysis: (1) DRM testing and counseling were valuable to clinical decision-making and reassuring to patients, with timely results allowing providers to change patient ART regimens faster; (2) providers and policymakers desired an amended and potentially decentralized DRM testing process that incorporates quicker sample-to-results turn-around-time, less burdensome procedures, and greater patient and provider “empowerment” to increase comfort with testing protocols; (3) facility-level delays, deriving from overworked facilities and sample tracking difficulties, were highlighted as areas for improvement.

**Conclusions:**

DRM testing has the potential to considerably improve patient health outcomes. Key informants recognized several obstacles to implementation and desired a more simplified, time-efficient, and potentially decentralized DRM testing process that builds provider comfort and confidence with DRM testing protocols. Further investigating the implementation, endurance, and effectiveness of DRM testing training is critical to addressing the barriers and areas of improvement highlighted in our study.

**Trial Registration:**

NCT03820323.

## Background

Kenya has one of the highest HIV prevalence rates in the world, approximately 4.2% in 2020 among adults, with an estimated 870,000 women living with HIV (WLHIV) and 82,500 children living with HIV (CLHIV) [1–3]. While 90% of WLHIV in Kenya receive antiretroviral therapy (ART) during their pregnancy, only an estimated 73% achieve viral suppression (VS) during the pregnancy and postpartum periods [1]. Lack of VS among pregnant WLHIV is one of the leading risk factors of vertical transmission of HIV, ultimately affecting pediatric HIV prevalence. Globally, only 40% of CLHIV had suppressed viral load (VL), compared to 67% of adults who had achieved VS [4]. Kenya has a high prevalence of pediatric HIV, with an estimated 111,500 CLHIV and 5,200 newly infected children under the age of 14 in 2020 [2].

While there are a variety of underlying causes for lower VS rates in both WLHIV and CLHIV, HIV drug resistance mutations (DRMs) account for some treatment failures [5]. DRM testing can greatly influence individual patient treatment decisions and represents the standard-of-care (SOC) in high-resource settings [6]. Routine surveillance of DRMs is also valuable in determining which ART regimens are selected as first or second-line treatment choices [7, 8]. DRMs have a substantial influence on the durability and longevity of existing ART regimens, particularly in the case of CLHIV, given their anticipated life-long use of the medications [9–11]. As most recently recommended by the Kenyan Ministry of Health (MOH), preferred first-line ART regimens for children and adults include dolutegravir. Prior to dolutegravir roll-out, the leading regimens included efavirenz and protease inhibitors [12].

International groups, including the Joint United Nations Programme on HIV/AIDS and the World Health Organization, have raised concerns regarding the rate at which HIV DRMs are increasing in resource-limited settings (RLS) [13, 14]. For example, in a survey from 10 sub-Saharan African countries, approximately half of infants newly diagnosed with HIV had drug-resistant strains before initiating ART [13]. Among the sampled patients in our parent study Opt4Kids (described in detail below), which included children enrolled in HIV care in western Kenya, 100% of the children with virologic failure had at least one DRM and 85% had at least one major DRM [15]. Similarly, in our parent Opt4Mamas study in pregnant and postpartum WLHIV, while rates of DRM were lower in women with virologic failure, DRMs identified provided critical information for rapid switch of ART to prevent HIV transmission during pregnancy and breastfeeding.

Recognizing the role HIV DRMs play in individual treatment determinations as well as for national policy guidance, many RLS have been scaling up DRM testing [16, 17]. However, scaling up DRM testing in such settings has been complex for various reasons, including procuring and maintaining expensive testing equipment, recruiting highly trained laboratory staff for molecular testing, developing and maintaining sample transport networks, and developing clinical capacity for use of such specialized testing [18, 19]. Several questions remain regarding DRM testing scale-up, including those related to patient understanding of DRM testing and results, provider comfort in interpretation and counseling of DRM testing results, and health systems optimization for wider use of DRM testing. To better understand how DRM testing can be implemented effectively, we conducted this qualitative sub-study within two parent studies. The goal of the parent studies was to evaluate the impact of point-of-care (POC) VL testing combined with targeted DRM testing and clinical decision support among CLHIV (Opt4Kids) and pregnant/postpartum WLHIV (Opt4Mamas). In this analysis, we aimed to better understand: (1) perceptions of DRM testing among patients, providers, and policymakers, (2) comfort in interpretation and counseling experiences among patients and providers, and (2) future improvements in the current DRM testing approach in western Kenya. To our knowledge, our study is the only qualitative investigation of CLHIV, pregnant WLHIV, and provider/policy maker perspectives on HIV DRM testing.

## Methods

### Study context, setting, and researcher positionality

The study was conducted in Kisumu County in western Kenya. Kisumu is the third largest city in Kenya with large agricultural industries that contribute largely to the broader East African economy [20]. Adult HIV prevalence is 17.5% in Kisumu, three times that of the national average, while pediatric HIV incidence is the second highest in the county [20–22]. The clinical facilities where the study was conducted were led by the implementing partner, Family AIDS Care and Education Services (FACES). Dr. Patrick Oyaro (a Kenyan principal investigator) and Dr. Lisa Abuogi (an American principal investigator), held leadership positions in FACES at some point in their lives and have developed strong connections with the County-level MOH. We conducted ad hoc meetings with the project team, which included academic researchers (including one primary investigator who belonged to the Kenyan community, one who belonged to the South Asian American immigrant community, and one who belonged to the White American community), study staff (including three who belonged to the Kenyan community, one who belonged to the Somali American immigrant community, and three who belonged to the Asian American/Pacific Islander community), and other stakeholders as needed to review our ongoing analysis and findings. Since many project team members were not of Kenyan background, the non-Kenyan members vetted the emerging themes and subthemes with our Kenyan team members to ensure the findings and interpretation resonated with their perceptions, beliefs, or lived experiences.

### Parent studies

Opt4Kids is an open-label, randomized controlled trial that examined the impact of POC VL testing in combination with targeted DRM testing and clinical decision guidance on VS among CLHIV on ART [15, 23]. The study recruited 704 CLHIV aged 1–14 years in five high-volume HIV treatment centers in Kisumu, Kenya, and followed them for 12 months. Study staff at each facility approached potential participants’ primary caregivers for study participation at routine clinical visits and obtained informed consent, with additional assent given by participants aged 13–14 years. Eligible children were randomly assigned to the intervention or SOC. The intervention included POC VL testing every 3 months using GeneXpert® technology, previously used in these facilities for tuberculosis diagnosis, targeted DRM testing for VL ≥ 1000 copies/mL, and clinical management support for the facility providers, including multidisciplinary case review (including facility providers and staff) for interpretation of the DRM testing results. The control or SOC arm followed national guidelines and included SOC VL testing every 6 months and approved DRM testing via a centralized approval process. Specifically, the NyaWest Technical Working Group reviewed and approved SOC DRM testing usually for those with virologic failure on a protease inhibitor-based first line or on second or third line ART who continued to have viremia after adherence optimization. The same committee provided guidance to providers on clinical management. DRM testing was conducted at a national reference laboratory.

Opt4Mamas is a prospective cohort study that compared VS rates pre- and post-implementation of a POC VL testing intervention among pregnant/postpartum WLHIV newly initiating or already on ART in the same five HIV treatment facilities. We enrolled 820 pregnant women during their antenatal care (ANC) visits and followed them for 6 months. During pre-intervention implementation, all enrolled women received SOC testing throughout pregnancy/postpartum care. During the post-intervention period, the study offered POC VL testing combined with targeted DRM testing and clinical decision guidance (as for Opt4Kids described above), and a new cohort of pregnant/postpartum women were enrolled to receive the intervention.

For both studies, a Clinical Management Committee (CMC) was developed for intervention group patients modelled after, and including some members of, the existing MOH clinical management technical working group in Kisumu County. The CMC was comprised of the head of the Kisumu-based Technical Working Group, clinical providers from the five facilities, study personnel and lead investigators, HIV specialists from the MOH and other countries, and technical advisers from HIV implementing partners. The committee conducted case reviews using a standardized form prepared by facility staff. It convened on a regular basis to discuss cases and to provide recommendations about ART regimen changes and case management using a study-developed standardized form.

### Study procedures

For the qualitative data collection for both studies, we conducted semi-structured in-depth interviews from December 2019 to December 2020 with six subgroups of key informants, including: (1) adolescent (ages 13 years and above) study participants for Opt4Kids, (2) caregivers of children enrolled in Opt4Kids, (3) women newly initiating ART during pregnancy in Opt4Mamas, (4) women already on ART with viremia at some point after enrollment in ANC in Opt4Mamas, (5) providers and other facility staff at our study sites, and (6) policymakers and other stakeholders at the local and national levels. Within the last two groups, we sampled individuals who worked in clinical care as well as in the laboratory section to ensure we had a wide breadth of perspectives. Study staff worked with the community partners and those attending community stakeholder meetings to help generate the initial pool of key informants for the key informant interviews (KIIs). We used convenience sampling to recruit patients already enrolled in the parent studies who came to the clinic during our interviewing period. Additionally, we utilized purposive sampling to recruit laboratory staff, facility staff, policymakers, and other stakeholders. Within each subgroup, we aimed to perform approximately 10–15 KIIs until saturation of themes was reached.

Our interview guides were designed using a socioecological model of VS, [24] which considers individual, interpersonal, organizational, and structural/policy variables affecting VS, with an additional emphasis on operational aspects of POC VL and DRM testing. The interview guides covered the following domains: (1) barriers and facilitators to ART usage and VS, (2) VL literacy and experiences with SOC VL and DRM testing in regular care, (3) experiences with POC VL and DRM testing within the study, and (4) how to scale up both SOC and POC DRM and VL testing for programmatic use. For this analysis we focused on DRM testing aspects of the KIIs; for findings relevant to VL testing, please reference a related publication [25].

### Data collection

We obtained written informed consent for all participants in the qualitative portion of the studies. Informed consent was obtained for minors from their primary caregivers, with additional assent given by participants aged 13–14 years. We collected de-identified socio-demographic information for key informants not already participating in our parent studies on a paper form, and later entered the information into a database. Our Kenyan study team members, who were research nurses or clinical officers, conducted the interviews and received centralized in-person training in interviewing techniques from the principal investigators. The interviewers had considerable prior experience conducting KIIs and focus group discussions with several studies conducted in the region. Of note, most of the facility staff, leadership, policymaker, and other stakeholder interviews were conducted by our team’s Kenyan research coordinator (EB). Interviews lasted 30–60 min, and field notes were taken during interviews. KIIs were conducted over video call via Zoom or in-person and audio-recorded in a private setting within the HIV treatment facilities. Interviews were performed in the participants’ chosen language, which was either English (largely for providers and stakeholders), Kiswahili, or Dholuo. Either the same interviewer or another member of the research staff transcribed the interviews directly into English if they occurred in Kiswahili or Dholuo. If the interviewer was not transcribing the recording personally, they reviewed the English transcription for accuracy, and any discrepancies were resolved by discussion among research staff members. RCP and EB read initial transcripts and provided feedback to each interviewer for iterative improvements in interviewing as well in the guides. English transcripts were then uploaded into NVivo (version 12.0, QRS International Pty Ltd.) for coding and analysis.

### Data analysis

We used inductive coding [26, 27], and three team members (SRQ, AJS, and SAH) carried out the coding under the supervision of RCP via weekly meetings and with iterative input from the larger research team, which included members from Kenya, via as-needed conference calls. SAH created an initial codebook based on KII guides and her read of the initial few transcripts, and then team members AJS, SRQ, and SAH iteratively modified the codebook as transcript coding progressed. Initially, two transcripts were coded collaboratively, in a group setting at the same time by all three coders, and then 1–2 transcripts were double-coded separately by coders, with any differences in coding addressed through consensus. The remaining transcripts were coded independently by various coders, with one coder (SAH) reviewing all transcripts’ coding in NVivo. We utilized thematic analysis to maintain an analytic codebook which organized our codes into overarching domains with subsequent themes, sub themes, and illustrative quotes, both convergent and divergent.

## Results

We conducted a total of 68 KIIs with CLHIV (*n* = 8), caregivers of participating CLHIV (*n* = 18), WLHIV who were newly initiating ART (*n* = 13), WLHIV who were already on ART with viremia (*n* = 10), HIV providers or laboratory staff (*n* = 6), facility-level leadership (*n* = 5), and county- or national-level policymakers (*n* = 8). Throughout this paper, the term “patients” refers to CLHIV, WLHIV who were newly initiating ART, WLHIV who were already on ART with viremia, and caregivers of participating CLHIV whom we interviewed. “Providers” includes HIV clinic providers or laboratory staff and facility-level leadership, and “policymakers” refers to county- or national-level policymakers whom we interviewed. Overall, while analyzing the attitudes and experiences with DRM testing among patients, providers, and policymakers three main themes emerged: (1) perceptions of DRM testing, (2) provider confidence and comfort with the DRM testing process, and (3) areas for future improvements. Table 1 provides notable quotations from the interviews, organized by the themes described below.

**Table 1:** Themes, subthemes, and quotes demonstrating attitudes towards and facilitators and barriers to DRM testing approaches

Main theme	Subtheme	Supporting Quote
Perceptions of DRM testing	Gaps in understanding of DRM testing among patients and providers	“No, they didn’t explain to me [the drugs resistance test results or anything to do with drug resistance].“ (35-year-old, female, caregiver)
		“I would wish to know more about drug-resistant testing…[m]aybe it was done but I don’t understand it.“ (Age unknown, female, adult patient)
		“I don’t routinely do the interpretation of [drug resistance tests]…I am not as comfortable with the interpretation of the result…many people with the facility may not even know what it is.” (Medical superintendent)
	Perceived benefits of DRM testing: informs clinical decision-making	“We got results just recently and we were told that further consultations are still being done to determine whether the child’s medications will be changed. They will give us a way forward.“ (56-year-old, female, caregiver)
		“[E]specially for the first line and second line, we actually just go blindly and sometimes you never know what the ART history has been, something that is never discussed very openly; as much as you may try to gather information, but sometimes people may be hiding certain issues; so, to me I feel it was giving us an aspect of making an informed decision.“ (County-level HIV and sexually transmitted infections coordinator).
	Perceived challenges of DRM testing: burdensome procedures	“For the standard-of-care it has been pathetic… it has been a challenge because there are so many channels of getting the test being done…before the test is being done, we have to get approvals, we have to discuss clients.” (Nursing officer)
		“[Before the DRM test,] we need to do a direct observation test but…maybe the client is somewhere where you cannot get…[y]ou find you [cannot know] whether the client has been taking the drugs or not.” (Clinic manger)
		“[T]here’s no clear roadmap of what you do next…[t]here’s a lot of mix-up and we really need to follow a lot for a DRM testing to be done. We need to do a lot of phone calls. So it’s not something that is easy for us to do.” (Clinic manager)
		“Currently, even the participants who are in need and the participants who are supposed to get that test actually don’t get that test because of a lot of bureaucratic layers…those are the aspects that need to be cleared.” (National-level lab specialist)
	Perceived challenges of DRM testing: lack of timeliness	“To me, I think [more timely results reception through the intervention] has really helped a lot and it has really helped us in decision-making for clients…we are really able to get those results early and act on them…the national [procedure] that takes like one month…[is] too much.” (Technical advisor)
		“[W]e have been having a challenge in terms of turnaround time or getting the results early…[t]he best thing with OptStudies is that…we are able to get the results very early.” (Clinic manager)
		“They really need to look at the turnaround of the DRT results because we are supposed to make decisions as early as possible. If [the tests] take a lot of time, it will then delay the process of intervention. So I would wish the process takes [a] shorted time…we should intervene as fast as possible because…the patient might be attacked by opportunistic disease and may end up dying before we even get the patients results.“ (Clinic manager)
		“[Sample-to-results turn-around time] should be timely and even the giving of the results to the client should not be long…because remember they are anxious, they are human beings too, they are failing and they have gone through sessions, they’re aware that they are failing. So getting the results back is also assurance or motivating. It lays the anxiety they might be having. And then they adjust very quickly to the changes and the possible ways of getting to suppression.“ (Nursing officer)
	Perceived challenges of DRM testing: facility-level challenges	“[W]e’ve also had the aspect where…they don’t track [the results] appropriately…one case that… I had to bring to their attention, then they found it in their system and it was actually just missed.” (County-level HIV and sexually transmitted infections coordinator)
		“[W]e have had delays because the facilities have so much in their hands; so, they have this case, then they say they will discuss the following day; so, they postpone the discussion. Then even when it has been discussed, summarizing the case is also another one. So, I would want to say that yes, we’ve had delays, and we’ve actually had backlogs for the DRM tests.” (County-level HIV and sexually transmitted infections coordinator)
	Perceived challenges of DRM testing: lack of funding	“[B]efore this I remember we had to ask for donations to do DRM testing for some participants…we were lucky that we had some well-wisher fund in the program, we call it the ‘participant fund’…for some participants, we actually had used the participant fund to facilitate…PMTCT [Prevention of mother-to-child transmission] women who needed.” (Technical advisor)
		“They are not done routinely not because they are harmful but because they are expensive. So, if it is done more frequently, it means it is affordable. So, making it affordable is another issue.“ (Medical superintendent)
Provider confidence and comfort with the DRM testing process	Overall confidence and comfort with DRM testing is limited among providers	“I have not met one [sample for DRM testing in my lab]…I don’t know if it’s because of the training or maybe they have been doing it and I [do not know]…whether they…desire to have the routine VL or DRM testing. But maybe empowerment should be done to [providers] so that they capture this.” (Lab coordinator)
		“[E]ither there is a knowledge gap that is making [providers] not request on this, or maybe there is fear of long turnaround time, or…maybe failure to read the guideline or that depth [of knowledge] to know that this is what I am supposed to do.” (County-level lab coordinator)
		“I don’t think we’ve empowered the providers to be able to actively do that and say…this is the [DRM] result that is back and it shows mutation for these and these” (Technical advisor)
	Trainings to increase confidence and comfort	“I have gone through advanced HIV clinical course, so I am confident and I also train others to interpret.“ (Technical advisor)
		“[T]raining is good because we cannot know everything. Maybe the way I interpret today, tomorrow the interpretation has changed because there’s so many researchers and medical knowledge is always changing. So having a training is good…[i]f we can be trained over the same then we will appreciate.“ (Nursing officer)
		We will really benefit more if we get training on it.“ (Clinic manager)
		“We’ve had several trainings, but they are still a bit shy when it comes to interpretation of the mutations and the significance of those mutations when you talk about drugs sensitivity and drug resistance.” (Technical advisor)
	Multidisciplinary discussions to increase confidence and comfort	“Most of the sites are doing a pretty good job [of interpreting DRM testing results] nowadays because of the NYAWEST-TWG…when they are discussing those cases, they’ve requested partners to support those health care workers to join these discussions. So people are getting better at doing it.” (Prevention of mother-to-child transmission technical advisor)
		“[My experience with the OptStudies] was good because I was also being put on board, discussing about the patients, making decision with different people, sharing ideas, sharing the challenges, the right ART regimen that the patient is supposed to be put on.” (Clinic manager)
Future Improvements	Decentralize the approval process	“Yes, if we could be doing everything in our lab then it could be better because, uh we take and then do it onsite and then the client gets his result immediately… [POC DRM testing is doable] if given the equipment because it is a matter of culture, it’s a matter of culture. What we need is just put uh, logistics in place… it doesn’t even need a lot.” (Lab incharge)
		“[U]sually we have a backlog of children who are not getting these tests, because we have this technical working group at the county level. So, if we decentralized this and give power to the facilities to recommend this test, maybe it would enable us to avoid the backlog.” (Technical advisor)
		“[F]or the decision of the DRM testing to be done, I feel that it should be right at the implementation phase and that is the service healthcare service providers because they are the one who identifies that this client is failing this regiment, they have the history of this clients right from initiation to the way they have walked on this journey with this client.” (Clinic manager)
	Facility-level improvements	“It should be made more accessible to more people, more facilities. For us, we want it even to be in the wards. To be accessible to people in the wards. So, then it makes it easier for us to make decisions in time.” (Medical superintendent)
		“[T]here is a big gap because either the sample was sent, six months down the line, the results page is still blank so you are like does it mean this result was not received or… so when you follow to the clinic, you find that maybe the results was received and maybe action was taken and yet at the lab register it is still blank. So, I think there is a lot of gaps there that need to be actually, considered to be taken some action so that we can.“ (County-level lab coordinator)
		“[Y]ou can get [the results] in the file, but it was filed there without their knowledge. So someone comes with a viral load results that is above 1000 and puts it in the file. Many clinicians are not good in flipping pages so they will not see it.” (Prevention of mother-to-child transmission technical advisor)
		“[F]or instance Lumumba has many clinicians who those case discussions would really make a difference in their lives in terms of even improving their skills and the knowledge on how to manage some of those clients because it’s fast hand with the consultant and everyone else and you know, the NYAWEST team is also part of that…[s]o it could be in a time where we can have as many as clinicians involved as possible to be looked at as an MDT with external facilities or something like that so that it is used to discuss the case but the same time to transfer skills and knowledge to as many as are available to be used.“ (Prevention of mother-to-child transmission technical advisor)
		“[W]e need to have also technical persons coming from different organizations and even the county, uh, to provide technical expert and advise on different clinical manifestation of participants and regimens switch or failures.“ (County director of public health)
	Build provider knowledge	“I think having an algorithm would help because for viral load, it’s pretty clear people are able to follow. So, I think that’s an area that uh, it’s a brilliant idea.” (County-level HIV and sexually transmitted infections coordinator)
		“They need to be trained. The training should be done so that even more of them…once they are not trained and many of them are not aware, they will not even request for it.” (Medical superintendent)
		“[C]apacity building of the clinical teams and even the diagnostic teams, I think it is an area that I can focus on so much and see how it can be done to support the class because I know there is resistance but we are missing opportunities.” (County-level lab coordinator)
	Build patient knowledge	“I would wish to know more about drug-resistant testing… [m]aybe it was done but I don’t understand it.“ (Age unknown, adult patient)
		“I can’t remember well [what was taught during sessions].“ (15-years-old, female, adolescent)
		“But now, the issue is the understanding is now what is uh, uh the question because really, to put it in a lay man’s language for them to be able to understand what you mean…the client will not be getting uh the right information.“ (County-level HIV and sexually transmitted infections coordinator)

### Perceptions of DRM testing

#### Gaps in understanding of DRM testing among patients and providers

Most patients reported a lack of awareness of DRM testing and had not previously experienced one being conducted for themselves or their children (for the caregivers). Notably, some patients indicated that clinic staff had not informed them that their samples were undergoing DRM testing. Patients reported only hearing about the DRM test results and next steps regarding their care after the results were back. In contrast, most providers reported familiarity with DRM testing through staff trainings, though some never received any formal training on DRM testing outside of the study. These providers expressed basic understanding of why and for whom DRM testing was conducted. However, these providers also acknowledged key limitations in in-depth understanding of DRM testing—such as knowing when to request a test, how to counsel patients based on their results, and how to make appropriate clinical decisions that adhere to the results—reporting that they had limited exposure to DRM testing or that they did not retain information they had previously received regarding DRM testing because their exposure to DRM testing outside of the study was so infrequent.

#### Perceived benefits of DRM testing: informs clinical decision-making

Providers/policymakers and patients stressed that DRM testing and DRM testing counseling were valuable to providers for optimal clinical decision-making and participants for reassurance, respectively. Study patients who had undergone DRM testing reported that the DRM testing results and counseling from the providers gave them helpful guidance or “a way forward” for next steps in their ART management. Specifically, DRM testing counseling provided patients with feedback on their adherence patterns and determined whether they needed a change in their ART regimen. As for providers, DRM testing results were crucial pieces of information that informed the clinical decision-making processes. One policymaker mentioned that clinical decisions were made easier with DRM testing because DRM testing provided insights into past antiretroviral (ARV) drug exposure for those few patients who, for whatever reasons, did not have prior ARV drug histories documented in their records. In such cases, providers felt empowered to determine appropriate treatment regimens, guided by actual information rather than blindly making a regimen change. Apart from their opinion that DRM testing aided clinical decision-making, providers and policymakers also appreciated the collaborative nature of the multidisciplinary discussions that occurred as part of the CMC meetings. Providers and policymakers stressed the importance of not having a single provider establish a patient’s treatment plan. Rather, collaborating on a team of varied and experienced staff boosted their confidence in the decisions made, especially for ART regimen changes.

#### Perceived challenges of DRM testing: burdensome procedures

Some of the perceived challenges stated by providers and policymakers included the difficulty of complying with current complicated SOC DRM testing protocols and requirements, such as mandatory bureaucratic approvals, multidisciplinary team discussions, and patient communication. As such, some providers commented that SOC DRM testing processes are “pathetic” and have been “disappointments”. Specifically, one provider mentioned that the two-week directly observed drug ingestion trial prior to requesting the DRM test can be challenging to achieve, due to difficulty contacting, coordinating with, and monitoring of patients for adequate completion of the trial requirements. The directly observed HIV treatment period involves a trained peer, such as a healthcare provider or family member, who observes the patient ingesting prescribed ART daily, until consistent adherence-enhancing behaviors are adopted. Many providers and policymakers emphasized that the bureaucratic steps involved with ordering and conducting a test were confusing and required close monitoring by providers, making the DRM testing process difficult to complete. As a result, the burdensome DRM testing process discouraged many clinicians from ordering test in the first place.

#### Perceived challenges of DRM testing: lack of timeliness

Another challenge stated by providers and policymakers was the overall delay associated with the SOC DRM testing process. Overwhelmingly, providers emphasized that mandatory bureaucratic processes and approvals delayed DRM testing procedures, due to the significant delays caused by the additional steps of enhanced adherence counseling summaries, directly observed therapy, and technical group discussions. During these delays, providers reported that patients’ overall health worsened with some patients developing opportunistic infections and, in one instance, one patient dying after waiting four months for test results. In contrast to the SOC protocols, many providers and policymakers perceived that timely reception of DRM testing results allowed providers to make clinical decisions and change patient ART regimens quickly.

In addition to providers and policymakers, patients also communicated dissatisfaction with the delays associated with the DRM testing process, desiring to know as soon as possible if their medication was working and if drug regimen changes were necessary. Patients, providers, and policymakers noted that timely reception of DRM testing results would decrease patient anxiety when waiting for results. As reported by providers, patients too felt that timely reception of DRM testing results was important to provide assurance and motivation to them.

##### Perceived challenges of DRM testing: facility-level challenges

In addition to the significant delays faced at the national level for DRM testing, providers and policymakers reported two facility-level challenges: (1) overworked facility staff and (2) difficulty tracking DRM testing results. First, several providers mentioned that DRM testing has been delayed because facilities have too many procedures to perform for the number of staff they have. Providers and policymakers commented that procedure backlog within clinics delayed testing procedures (such as the postponement of multidisciplinary team discussions), clinical decision-making, and patient care. Secondly, providers and policymakers commented on how the mishandling of DRM testing results, such as logistical issues of tracking DRM testing results, led to missed results and delayed care.

#### Perceived challenges of DRM testing: lack of funding

Lastly, providers stated that lack of funding is a significant challenge to DRM testing. Several providers emphasized that while the national HIV program covers the cost of the DRM test itself and is free to the clinics and patients, in certain cases, clinicians were only able to conduct DRM testing after requesting and receiving donations from FACES for laboratory tests not otherwise covered by the MOH. Providers highlighted that the high cost of the test prevented clinicians from ordering tests, making the test, as they stated, less accessible.

### Provider confidence and comfort with the DRM testing process

#### Overall confidence and comfort with DRM testing is limited among providers

Overall, the general lack of knowledge and formal training on when and how to order a test for the current national SOC process deterred many providers from requesting a test. One provider mentioned that their lab had never encountered a sample that qualified for DRM testing, acknowledging that providers were unaware of what qualified for further DRM testing.

#### Trainings to increase confidence and comfort

By and large, trainings were considered valuable to increase providers’ confidence to interpret DRM testing results and improve providers’ ability to teach others to interpret DRM testing results. One provider underscored that the absence of any type of DRM testing training at their clinic was the reason for their co-workers’ inability to interpret DRM testing results independently. At the same time, one policymaker noted that regardless of training reception, providers were still “shy,” lacking the confidence to correctly interpret results and make subsequent clinical decisions. Notably, despite not having undergone formal training to interpret testing results, several providers commented on how clearly laid out words such as “resistance” and “non-susceptible”, i.e., phenotypic interpretation using the Stanford HIV Drug Resistance Database, printed on DRM testing results documents by the resulting lab made DRM testing results interpretation easier. In addition to ordering and interpreting DRM testing results, providers also reported a lack of formal training on how to counsel patients after receiving and processing the DRM testing results themselves. One policymaker further highlighted that counseling patients can be difficult due to the challenge of making technical language around DRM testing more accessible for patients. On the other hand, when asked about the meaning of their DRM testing results, patients used non-technical and digestible language, such as “not working well” or “not effective,” to explain their results. Overall, most patients seemed to be satisfied by the language used by their providers to counsel on their results, expressing gratitude towards their provider’s kindness, guidance, and efforts to motivate them and alleviate their fears during these sessions, while desiring to know why they may have to change their drug regimens. Notably, patients trusted providers and study staff in their knowledge of HIV treatment, with two patients reporting that they wanted doctors and the study staff to educate and counsel them on their results.

#### Multidisciplinary discussions to increase confidence and comfort

Lastly, several providers commented that the multidisciplinary discussions hosted by the study markedly improved providers’ abilities to interpret DRM testing results, giving them opportunities to gather differing perspectives on which ART regimen was optimal for a patient. Providers and policymakers noted that incorporating a wider range of stakeholders from different facilities, organizations, and counties into the multidisciplinary discussions would allow for more enriched discussions, improving their DRM testing interpretation skills.

### Future improvements

#### Decentralize the approval process

Several providers and policymakers wanted to decentralize the overall DRM testing ordering process to decrease the number of bureaucratic layers, which would not only better empower local facilities to seek out DRM testing but also decrease turn-around time and expedite clinical decision-making process. Multiple providers and policymakers suggested allowing providers to request a test without the approval of the multidisciplinary discussion team which would prevent a backlog of DRM testing orders. Nonetheless, providers and policymakers suggested that developing carefully considered and planned national algorithms would help ensure the implementation of a decentralized DRM testing ordering process optimized on the limited resources for such testing.

#### Facility-level improvements

Providers and policymakers identified three facility-level improvements: (1) increasing clinic capacity for DRM testing on-site via POC assays, (2) improving systems for tracking the return of results, and (3) incorporating more diverse voices into multidisciplinary discussions.

First, providers and policymakers suggested increasing testing capacity by making POC DRM testing available on-site. Although providers recognized that this would involve increasing human capacity and acquiring equipment, one provider emphasized that this is a matter of changing culture and would require minimal effort. Other providers commented that having DRM testing technologies on-site would allow providers to decide more quickly which regimens to administer to a patient.

Second, providers and policymakers desired a system that tracks DRM testing results within each facility. One policymaker, for example, expressed concern over the misfiling of DRM testing results which had resulted in delayed modification of treatment plans for patients and delayed care. One lab coordinator expressed confusion as to whether results from the lab were received by the facility or not, expressing desire to improve systems for tracking the return of DRM testing results from the lab to the facility.

Third, providers and policymakers strongly approved of the collaborative multidisciplinary team discussions within the studies, advocating for the expansion and diversification of these discussions to include clinicians and technicians from different facilities. One policymaker believed this would allow for a greater exchange of skills, knowledge, improve case management, and patient care coordination. Providers and policymakers also expressed interest in holding regular multidisciplinary discussions, on a monthly basis (they were held ad hoc during study follow-up depending on case volumes).

#### Build provider knowledge

Additionally, providers and policymakers expressed great interest in the provision of trainings to providers so that they are aware of and feel empowered to order tests, interpret DRM testing results, counsel patients appropriately, and make subsequent clinical decisions. Notably, one provider emphasized that because DRM testing is not routinely conducted, many providers are unaware of when to order a test, which has led to missed opportunities for testing. Providers mentioned that having regular, up to date trainings would increase the confidence and comfort of providers to order these tests. Providers noted that trainings should also dissuade providers’ fear of erroneously ordering a test when not indicated, especially layered onto a backdrop of limited resources for such testing in the first place. Overall, providers and policymakers expressed great interest in the creation of an algorithm that gives providers clear directions on when to order a DRM test.

#### Build patient knowledge

Patients and providers highlighted the need to increase patient knowledge of DRM testing. Specifically, providers emphasized that patients need clearer messaging on how the DRM testing process works. Those providers who had counseled patients on DRM testing results reported that they tended to focus counseling on what the DRM testing results meant for the patient, namely whether the patient needed to change ART regimens or adherence habits, rather than detailing specific DRM testing procedures and processes. Interestingly, we found that patients wanted to learn more about the details behind the DRM testing procedures, not just the end implications of the DRM testing result, although some patients who were given DRM testing training often could not recall any information that they learned. For instance, one patient with high VL was aware that her blood sample was collected, but she was confused as to what the blood test was specifically measuring.

## Discussion

By qualitatively exploring perceived attitudes towards, facilitators to, and barriers to current DRM testing approaches in Kenya among patients, providers, and policymakers, our study provides key insights on how to leverage existing DRM testing procedures for more optimal scale-up in RLS. The perceived potential for DRM testing to significantly impact patient health outcomes, i.e., resulting in an ART regimen change, was supported by all, from patients to policymakers. Improving provider confidence and comfort with ordering and interpreting DRM testing results via regular trainings and implementing a simpler, decentralized DRM testing ordering process are clear next steps in improving DRM testing scale-up in Kenya and related settings.

Globally, HIV drug resistance is a substantial threat to the success of ART and the elimination of HIV [7]. Within RLS, the limited availability of ARV drug options, high cost of second- and third-line regimens, and limited capital (e.g., laboratory equipment, personnel, maintenance costs) and high-quality laboratory infrastructure for timely delivery of accurate DRM testing results illustrate how crucial it is that ART regimens are used optimally, both in switching patients whose ART is failing and not switching patients whose drug resistance to the current regimen is not the reason for virologic failure [28]. With transmitted or acquired HIV drug resistance only increasing as more people have access to ART and age on their current regimens, the fundamental next questions for the global HIV community are how to make DRM testing more accessible to patients and providers in RLS.

DRM testing was perceived as fundamental to the clinical decision-making process for patients with virologic failure, by allowing providers to infer past ARV drug exposures when missing from records or not disclosed by patients, and to develop a plan for optimal ART regimens moving forward. For example, a study conducted in South Africa, reported that genotypic resistance testing informed clinical decision-making, causing providers to select a second-line regiment different to what would have been originally selected by the provider [29, 30]. DRM testing also prevents premature switches to costly or unnecessary empiric regimens, potentially increasing overall cost-efficiency and limiting any patient side effects or adverse events that may occur when changing drug regimens [18]. In addition to informing clinical decision-making, the presence of DRM testing services within clinical settings raises awareness among clinical providers and patients of issues relating to ARV resistance and treatment failure—issues that may be otherwise unknown [30]. That is, the process of reviewing DRM testing results reports served as educational tools by exposing nurses, clinicians, counsellors, and patients to drug resistance patterns within clinical settings [30]. Nonetheless, incorporating more frequent DRM testing among patients with virologic failure on first- or second-line ART, when used in combination with POC VL testing and clinical decision-making support, did not improve VS among all children enrolled in our Opt4Kids study. However, a subset of children who did require a regimen change did show a statistically significant rate of VS [31, 32]. Similarly, a study conducted in public-sector HIV clinics in Uganda and South Africa found that incorporating DRM testing into the SOC procedures for patients whose first-line regimens failed did not improve patients’ VS rates after 9 months, findings that are consistent with other studies that suggest limited short-term and long-term virologic benefits of DRM testing [33–35]. These findings, however, are in conflict with other studies that report stated benefits of DRM testing on virologic outcomes [36]. Overall, further investigation into the impact of DRM testing will be essential to guiding selection of second- and third-line regimens, managing treatment failure, coordinating patient care, and improving health outcomes.

While the DRM testing results were viewed as informative clinical tools, bureaucratic regulations were repeatedly emphasized as obstacles to optimal patient care, slowing down the DRM testing process and complicating DRM testing procedures. Our patients highlighted that the lack of timely receipt of results, a well-reported challenge for DRM testing, increased patient anxiety over delayed results and perceived worsening of patient health during the wait [13, 18]. Slow and burdensome administrative procedures, such as mandatory consultations, intensive adherence counseling sessions, directly observed therapy, and coordination of multidisciplinary team discussions, resulted in a backlog of treatment failure cases, deterred providers from ordering tests, and confused providers who followed patients’ results through the treatment cascade. To tailor service provision and facilitate the DRM testing process, providers and policymakers suggested decentralizing DRM testing procedures or transferring management and decision-making power to county- level or even to local facilities. Admittedly, when resources are scarce for DRM testing, it is understandable that tension exists between enabling a decentralized DRM testing ordering process and ensuring optimal use of the testing, but this has been done successfully in South Africa and Botswana [29, 30]. Moreover, a decentralized process could be facilitated by POC, or even near POC DRM testing, a simpler-to-operate POC technology that provides on-site results return within health facilities. Despite potential positives of decentralized DRM testing, we acknowledge that decentralization of DRM testing has been criticized for its high costs and impracticality within constrained clinic settings, leading others to suggest building upon existing capacities by strengthening centralized high-throughput laboratories to address issues surrounding sample referral and results delivery [18, 29]. How to optimally decentralize DRM testing procedures in RLS is a key area for further research.

In addition to bureaucratic regulations, the overall lack of provider and patient knowledge of the DRM testing process was another reported challenge. Specifically, the confusion among providers of when and how to order and interpret tests, as well as the lack of confidence in their recommendations to change ART regimens, remained a barrier to DRM testing use, delaying patient care and preventing patients in need of DRM testing from undergoing it—barriers that have also been reported elsewhere [37]. The introduction and implementation of up-to-date DRM testing training programs presents an opportunity to address the lack of provider knowledge, confidence, and comfort in ordering and interpreting tests. Expanding clinic-level healthcare workers’ capacity to order, interpret, and counsel on DRM testing is critical due to the scarcity of specialist clinicians and the overall issue of drug resistance becoming a growing concern in RLS [29]. Exploring ways to effectively implement these trainings and ensure their success will be key in improving providers’ fundamental ability to comprehend, follow, and operate under national protocols. Furthermore, the lack of patient knowledge limited patients’ involvement in understanding and managing their own health. Our data suggests that patients were eager to receive messaging regarding what DRM testing entails and how the findings reflect their adherence; national programs will need to develop training tools for providers to better enable them to educate and counsel their patients as well. Overall, increasing access to knowledge about DRM testing will equip clinicians with tools to provide more effective care and provide patients with knowledge to better understand their own health.

Other challenges to DRM testing implementation in RLS include high capital expenditures and test costs, limited laboratory infrastructure, lack of skilled staff, and issues surrounding specimen collection, handling, tracking, and transport to centralized laboratories—findings that have been reported elsewhere [18, 29, 38]. Despite these barriers, new types of DRM testing technologies have significantly lowered cost and increased access to testing in RLS [18, 29, 37]. Feasible technologies to detect DRMs include sequencing-based assays, such as Sanger and next-generation sequencing in centralized laboratories, but coordination of HIV care could potentially be more efficient and effective with rapid, economical, and simpler testing technology [15, 22]. Although it is anticipated that widely validated Sanger assays will continue to play a primary role in DRM testing in RLS, point-mutation assays and genotype-free prediction systems offer the possibility of decentralization through POC or near-POC use [18]. For instance, in comparison to Sanger sequencing assays, tests such as ‘OLA-Simple’, a near-POC test based on the oligonucleotide ligation assay (OLA), have faster turnaround times from sample-to-results and improved sensitivity, while requiring less expensive equipment and less complex workflows [39]. We are currently attempting to implement a field validation study of OLA Simple for our study samples in Kenya, [40, 41] and the same team is in the midst of developing a POC assay that detects HIV VL and, amongst those with a certain threshold of VL, automatically conducts DRM testing [42]. Such types of POC DRM testing, which several providers within our study advocated for, have the potential to bypass delays associated with centralized procedures, reduce chances for specimen mishandling, and decrease the risk of losing patients to follow-up for clinic visits [37]. Coupling VL testing and DRM testing facilitates earlier entry of a patient into the treatment cascade prior to the acquirement of further DRMs, avoids high costs from premature switches to second or third-line therapy, and maximizes durability of ART options within RLS [7, 13, 18, 28, 43, 44]. Looking forward, coupling POC DRM testing with routine POC VL testing can inform providers within a single visit if patients need to switch regimens, potentially improving VS [37].

While our qualitative study sheds light on how impactful DRM testing might be for optimal HIV treatment decisions and current challenges in operationalizing DRM testing in Kenya, it also has several limitations. We were unable to obtain meaningful interviews from our young adolescent participants (ages 13–14 years), so we were unable to include the perspectives that may be unique to this population. We also did not sample providers based on years of experience; hence, our analysis does not establish connections between years of experience and comfort in use of DRM testing. Furthermore, the transcript coding and initial analysis was not led by Kenya-based team members, so our analytic framework may lack the necessary context to adequately interpret interviews and make appropriate conclusions. While the Seattle-based coding and analysis team met on several occasions with the Kenya-based team, a coding and analysis effort entirely led by Kenya-based team members could have possibly resulted in different conclusions or different emphases on certain findings. Additionally, our investigation was conducted during the COVID-19 pandemic, which made it difficult to conduct in-person interviews due to limitations in travel, especially with national-level stakeholders, which may have potentially influenced the responses we elicited, though we still captured body language via video calls in Zoom. Though our patient and provider sampling was limited to the facilities where the parent studies took place in Kisumu, Kenya, the themes we presented are applicable to other clinics in Kenya and likely other RLS. Notwithstanding these limitations, our work presents some of the most detailed findings to date with implications for DRM testing scale-up in RLS.

## Conclusions

Our study reveals that patients, providers, and policymakers perceived DRM testing offering great value in enabling them to make optimal treatment decisions. However, current DRM testing processes, such as the study setting in Kenya, face considerable obstacles to wider implementation in RLS, including a need for a simplified, more time-efficient, and potentially decentralized DRM testing process that does not undermine provider confidence and comfort ordering and interpreting DRM testing results. As scale-up of DRM testing is increasing, with both conventional and novel POC assays, it is time for national HIV treatment programs, supported by international agencies, to develop enhanced provider training that fosters confidence in ordering DRM testing and interpreting subsequent results and to create messaging for patients to bolster their understanding of DRM testing. Further investigation into creating effective methods for implementing and ensuring the effectiveness of these trainings and messaging strategies are needed soon, as global increases in drug resistance will only become a more widespread issue over time.

## Data Availability

The corresponding author will allow sharing of de-identified data, data codebook, and other data elements upon request and ethics approval, and upon submission of relevant documents, such as research protocol, and signed data access agreement.

## References

[1] UNAIDS. Country progress report - Kenya Global AIDS Monitoring 2019. Preventing mother-to-child transmission of HIV, Kenya. 2019;2.3. https://www.unaids.org/sites/default/files/country/documents/KEN_2019_countryreport.pdf.

[2] Joint United Nations Programme on HIV/AIDS. UNAIDS Kenya-HIV and AIDS estimates. Published online 2020. https://www.unaids.org/en/regionscountries/countries/kenya.

[3] GHO | By category | Prevalence of HIV among adults aged 15 to 49 - Estimates by WHO region. WHO. https://apps.who.int/gho/data/node.main.622?lang=en. Accessed 9 June 2023.

[4] UNAIDS. Co-creating a new global initiative to end AIDS among children, adolescents and their mothers. Published online 2021. https://www.unaids.org/en/resources/presscentre/featurestories/2021/december/20211210_icasa-pmtct.

[5] Pilgrim NA, Okal J, Matheka J, Mukui I, Kalibala S (2018). Challenges to and opportunities for the adoption and routine use of early warning indicators to monitor pediatric HIV drug resistance in Kenya. BMC Pediatr.

[6] World Health Organizations (WHO). Consolidated guidelines on HIV prevention, testing, treatment, service delivery and monitoring: recommendations for a public health approach. 2021. https://www.who.int/publications/i/item/9789240031593. 34370423

[7] Clutter DS, Jordan MR, Bertagnolio S, Shafer RW (2016). HIV-1 drug resistance and resistance testing. Infect Genet Evol.

[8] Takou D, Fokam J, Teto G (2019). HIV-1 drug resistance testing is essential for heavily-treated patients switching from first- to second-line regimens in resource-limited settings: evidence from routine clinical practice in Cameroon. BMC Infect Dis.

[9] Boender TS, Hoenderboom BM, Sigaloff KC (2015). Pretreatment HIV drug resistance increases regimen switches in sub-Saharan Africa. Clin Infect Dis.

[10] Ndashimye E, Arts EJ (2019). The urgent need for more potent antiretroviral therapy in low-income countries to achieve UNAIDS 90-90-90 and complete eradication of AIDS by 2030. Infect Poverty.

[11] Rutstein SE, Golin CE, Wheeler SB (2016). On the front line of HIV virological monitoring: barriers and facilitators from a provider perspective in resource-limited settings. AIDS Care.

[12] National AIDS & STI Control Programme (NASCOP). Kenya HIV prevention and treatment guidelines 2022. https://app.box.com/s/3ox5vm885qamwloah6sciljvmxcle4oq.

[13] World Health Organization. Global action plan on HIV drug resistance: 2017-2021. https://www.who.int/publications/i/item/978-92-4-151284-8.

[14] UNAIDS. Understanding fast-track: accelerating action to end the AIDS epidemic by 2030. https://www.unaids.org/sites/default/files/media_asset/201506_JC2743_Understanding_FastTrack_en.pdf.

[15] Patel RC, Oyaro P, Thomas K, Basha G, Wagude K, Mukui I, et al. Impact of point-of-care HIV viral load and targeted drug resistance mutation testing on viral suppression among Kenyan children on antiretroviral therapy: Results from an open-label, randomized controlled trial (Opt4Kids). J Int AIDS Soc. Under Review 2023.

[16] Nasir IA, Emeribe AU, Ojeamiren I, Aderinsayo Adekola H (2017). Human immunodeficiency virus resistance testing technologies and their applicability in resource-limited settings of Africa. Infect Auckl.

[17] Inzaule SC, Osi SJ (2018). High prevalence of HIV drug resistance among newly diagnosed infants aged < 18 months: results from a nationwide surveillance in Nigeria. J Acquir Immune Defic Syndr.

[18] Inzaule SC, Ondoa P, Peter T (2016). Affordable HIV drug-resistance testing for monitoring of antiretroviral therapy in sub-Saharan Africa. Lancet Infect Dis.

[19] Duarte HA (2018). Implementation of a point mutation assay for HIV drug resistance testing in Kenya. AIDS Lond Engl.

[20] Kenya Ministry of Health-Council of Governors. County government of Kisumu county gender data sheet. Published online July 2019.

[21] National AIDS and STI Control Programme (NASCOP). Preliminary KENPHIA 2018 report Kenya ministry of health, Nairobi, Kenya. Accessed 2023. https://phia.icap.columbia.edu/wp-content/uploads/2020/04/KENPHIA-2018_Preliminary-Report_final-web.pdf.

[22] Siaya County Report on the HIV Implementing Partners Online Reporting System (HIPORS). Maisha! National AIDS Control Council; For the financial year 2016/2017. https://nsdcc.go.ke/wp-content/uploads/2018/04/Siaya.pdf.

[23] Patel RC (2020). Optimizing viral load suppression in Kenyan children on antiretroviral therapy (Opt4Kids). Contemp Clin Trials Commun.

[24] McLeroy KR, Bibeau D, Steckler A, Glanz K (1988). An ecological perspective on health promotion programs. Health Educ Q.

[25] Qian SR, Hassan SA (2022). “After viral load testing, I get my results so I get to know which path my life is taking me”: qualitative insights on routine centralized and point-of-care viral load testing in western Kenya from the Opt4Kids and Opt4Mamas studies. BMC Health Serv Rsch..

[26] Braun V, Clarke V (2014). What can “thematic analysis” offer health and wellbeing researchers?. Int J Qual Stud Health Well-Being.

[27] Braun V, Clarke V (2006). Using thematic analysis in psychology. Qual Res Psychol.

[28] Johnston V, Fielding KL, Charalambous S, Churchyard G, Phillips A, Grant AD (2012). Outcomes following virological failure and predictors of switching to second-line antiretroviral therapy in a South African treatment program. JAIDS J Acquir Immune Defic Syndr.

[29] Lessells RJ, Avalos A, de Oliveira T (2013). Implementing HIV-1 genotypic resistance testing in antiretroviral therapy programs in Africa: needs, opportunities, and challenges. AIDS Rev.

[30] Lessells RJ, Stott KE, Manasa J (2014). Implementing antiretroviral resistance testing in a primary health care HIV treatment programme in rural KwaZulu-Natal, South Africa: early experiences, achievements and challenges. BMC Health Serv Res.

[31] Patel RC, Oyaro P, Thomas K, Basha G, Wagude K, Mukui I, et al. Impact of point-of-care HIV viral load testing in Kenyan children: a randomized trial. Conference on Retroviruses Opportunistic Infections, abstract 33, 2022. CROI 2022. https://www.croiconference.org/croi-2022/.

[32] Abuogi L, et al. High drug resistance and need for ART change in children with viral failure in Kenya. Conference on Retroviruses and Opportunistic Infections, abstract 722, CROI 2022. https://www.croiconference.org/croi-2022/.

[33] Siedner MJ, Moosa MS, McCluskey S, Gilbert RF, Pillay S, Aturinda I, et al. Resistance Testing for Management of HIV Virologic Failure in Sub-Saharan Africa : An Unblinded Randomized Controlled Trial. Ann Intern Med. 2021;174(12):1683–92. 10.7326/M21-2229. Epub 2021 Oct 26. PMID: 34698502; PMCID: PMC8688215. 10.7326/M21-2229 PMC8688215 34698502

[34] Wegner SA, Wallace MR, Aronson NE (2004). Long-term efficacy of routine access to antiretroviral-resistance testing in HIV type 1–Infected patients: results of the clinical efficacy of resistance testing trial. Clin Infect Dis.

[35] Panidou ET, Trikalinos TA, Ioannidis JP (2004). Limited benefit of antiretroviral resistance testing in treatment-experienced patients: a meta-analysis. AIDS.

[36] Tural C (2002). Clinical utility of HIV-1 genotyping and expert advice: the Havana trial. AIDS Lond Engl.

[37] Rhee SY, Jordan MR, Raizes E (2015). HIV-1 drug resistance mutations: potential applications for point-of-care genotypic resistance testing. PLoS ONE.

[38] Hermans LE, Steegen K (2020). Drug level testing as a strategy to determine eligibility for drug resistance testing after failure of ART: a retrospective analysis of South African adult patients on second-line ART. J Int AIDS Soc.

[39] Panpradist N, Beck IA, Ruth PS (2020). Near point-of-care, point-mutation test to detect drug resistance in HIV-1: a validation study in a Mexican cohort. AIDS.

[40] NIH RePORTer. Optimizing viral suppression for pregnant and postpartum women living with HIV through HIV resistance testing (Opt4Mamas) grant site. https://reporter.nih.gov/search/zbwDXyM3Hki0QIF7dVsBRQ/project-details/9844145.

[41] Thrasher Research Fund, Using novel point-of-care HIV drug resistance testing to optimize viral suppression rates among children living with HIV (Opt4Kidsplus). https://www.thrasherresearch.org/grant/15240?lang=eng.

[42] NIH RePORTER. V-OLA: point-of-care HIV viral load monitoring and drug resistance testing. https://reporter.nih.gov/search/a0M1_UbCWECXiLEXVXQiww/project-details/9844703.

[43] Hamers RL, Wallis CL, Kityo C, Siwale M, Mandaliya K, Conradie F, et al. PharmAccess African Studies to Evaluate Resistance (PASER). HIV-1 drug resistance in antiretroviral-naive individuals in sub-Saharan Africa after rollout of antiretroviral therapy: a multicentre observational study. Lancet Infect Dis. 2011;11(10):750–9. 10.1016/S1473-3099(11)70149-9. Epub 2011 Jul 27. PMID: 21802367. 10.1016/S1473-3099(11)70149-9 21802367

[44] Bonner K, Mezochow A, Roberts T, Ford N, Cohn J (2013). Viral load monitoring as a tool to reinforce adherence: a systematic review. JAIDS J Acquir Immune Defic Syndr.

